# Liquid biopsy for human cancer: cancer screening, monitoring, and treatment

**DOI:** 10.1002/mco2.564

**Published:** 2024-05-28

**Authors:** Hao Wang, Yi Zhang, Hao Zhang, Hui Cao, Jinning Mao, Xinxin Chen, Liangchi Wang, Nan Zhang, Peng Luo, Ji Xue, Xiaoya Qi, Xiancheng Dong, Guodong Liu, Quan Cheng

**Affiliations:** ^1^ Department of Neurosurgery The Second Affiliated Hospital, Chongqing Medical University Chongqing China; ^2^ Department of Psychiatry The School of Clinical Medicine, Hunan University of Chinese Medicine Changsha China; ^3^ Department of Psychiatry Brain Hospital of Hunan Province (The Second People’s Hospital of Hunan Province) Changsha China; ^4^ Health Management Center The Second Affiliated Hospital, Chongqing Medical University Chongqing China; ^5^ Department of Neurosurgery Fengdu People's Hospital, Chongqing Chongqing China; ^6^ College of Life Science and Technology Huazhong University of Science and Technology Wuhan China; ^7^ Department of Oncology Zhujiang Hospital, Southern Medical University Guangzhou China; ^8^ Department of Neurosurgery Traditional Chinese Medicine Hospital Dianjiang Chongqing Chongqing China; ^9^ Department of Cerebrovascular Diseases Dazhou Central Hospital Sichuan China; ^10^ Department of Neurosurgery Xiangya Hospital, Central South University Changsha China; ^11^ National Clinical Research Center for Geriatric Disorders Xiangya Hospital, Central South University Changsha China

**Keywords:** Circulating tumor cell, Circulating tumor DNA, Exosomes, ICI, Immunotherapy, Liquid biopsy, Noninvasive tumor detection, PD‐L1, Tumor‐related circulating cells

## Abstract

Currently, tumor treatment modalities such as immunotherapy and targeted therapy have more stringent requirements for obtaining tumor growth information and require more accurate and easy‐to‐operate tumor information detection methods. Compared with traditional tissue biopsy, liquid biopsy is a novel, minimally invasive, real‐time detection tool for detecting information directly or indirectly released by tumors in human body fluids, which is more suitable for the requirements of new tumor treatment modalities. Liquid biopsy has not been widely used in clinical practice, and there are fewer reviews of related clinical applications. This review summarizes the clinical applications of liquid biopsy components (e.g., circulating tumor cells, circulating tumor DNA, extracellular vesicles, etc.) in tumorigenesis and progression. This includes the development process and detection techniques of liquid biopsies, early screening of tumors, tumor growth detection, and guiding therapeutic strategies (liquid biopsy‐based personalized medicine and prediction of treatment response). Finally, the current challenges and future directions for clinical applications of liquid biopsy are proposed. In sum, this review will inspire more researchers to use liquid biopsy technology to promote the realization of individualized therapy, improve the efficacy of tumor therapy, and provide better therapeutic options for tumor patients.

## Graphical abstract

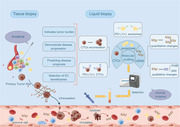

## 1 INTRODUCTION

At present, the threat of malignant tumors to human health is becoming more and more serious. Early diagnosis, growth monitoring, and therapeutic effects of malignant tumors are the keys to solving this problem.^1^ Various new treatment strategies have emerged, and higher requirements have been put forward for the current detection methods. There is an urgent need for more convenient, stable, and reliable tumor markers to provide tumor‐related information. The emergence of liquid biopsy technology can better deal with this problem. This review will briefly elaborate on the application of liquid biopsy in early tumor screening, tumor monitoring, and tumor treatment.^2^

Immunotherapy has revolutionized the treatment of tumors and opened up new avenues for treating advanced malignant tumors. However, its effectiveness in many populations still needs to be greatly improved.^3^ Since the first approval of immune checkpoint inhibitors (ICIs) for unresectable malignant melanoma, several different tumor types have benefited from United States Food and Drug Administration (US FDA)‐approved therapeutic indications regarding ICIs, and applications in this area are rapidly evolving.^4^, ^5^ However, the effect of immunotherapy is not effective in all populations, and according to clinical data, only about one‐third of patients show good treatment response in tumor immunotherapy.^6^, ^7^, ^8^ How to efficiently and accurately screen cancer populations that are validated for immunotherapy is currently an essential research direction to prolong the survival of these patients. There is a need for effective and stronger validation tools or biomarkers as indicators of the applicability of immunotherapy in the clinical.^9^, ^10^

Besides, targeted therapies have led to some significant advances in cancer treatment. Some targeted drugs have been successfully used in specific cancer types, such as HER2‐positive breast cancer and EGFR‐mutated non‐small cell lung cancer.^11^, ^12^ These drugs can more precisely intervene in the growth and division of cancer cells, causing less damage to normal cells than traditional radiotherapy and chemotherapy, thereby reducing side effects.^13^, ^14^ However, targeted therapy still faces some challenges and difficulties. First, some patients may develop drug resistance after receiving targeted therapy, resulting in weakened or ineffective treatment effects.^15^ Second, biopsy specimens are not easy to obtain.^16^ Targeted therapy is usually based on the molecular characteristics of the disease, and molecular information about the tumor needs to be obtained and is only available to patients with specific molecular changes. This limits the scope of the application of targeted therapy.^17^ Third, since the molecular characteristics of each patient may differ, the effect of the same targeted therapy may vary. Overall, targeted therapy has achieved some clinical success, but continuous research and efforts are still needed to solve the above challenges to improve the effect of treatment and expand its scope of application.^18^ In other words, there is also a need for effective and stronger validation tools or biomarkers as indicators of the applicability of targeted therapy in the clinical.

Liquid biopsy, as a novel technique, compensates for this current shortcoming. Human body fluids are a highly complex microenvironment that contains information far beyond the boundaries of human exploration. Getting information from body fluids has become the most recent trend. The development of liquid biopsies did not go smoothly due to various factors, such as a lack of thorough recognition of such abnormal molecules and immature extraction techniques. Lo et al. reported examining fetal DNA in maternal serum and plasma, showing that 10−15% of cfDNA in maternal plasma was released from the placenta.^19^ Noninvasive liquid biopsy methods have long been used to extract and analyze autosomal fetal aneuploidy in noninvasive prenatal testing in 1997.^20^ This operation pioneered the introduction of liquid biopsy into clinical application. However, molecules and cells containing tumor information in the body fluids of cancer patients have been continuously demonstrated to be present earlier. For example, circulating tumor cells (CTCs) were detected in 1868, and distributing free DNA was disclosed in 1948, which laid a firm foundation for applying liquid biopsy.^21^, ^22^, ^23^, ^24^, ^25^ Not until recent years have tremendous advances in detection technology reopened the eyes to the enormous potential of liquid biopsies. Many research programs have invested more in liquid biopsy technology, leading to its rapid development. Liquid biopsy is a noninvasive diagnostic technology that analyzes biomarkers in body fluids(blood, urine,^21^ saliva,^22^ pleural effusions,^23^ and cerebrospinal fluid (CSF),^24^ as well as stool,^25^ such as CTCs,^26^ circulating tumor DNA (ctDNA),^27^ circulating cell‐free RNA (noncoding and messenger RNA),^28^ extracellular vesicles (EVs),^29^ tumor education platelets,^30^ and so on, to obtain information about the disease status. Liquid biopsies have more potential for early cancer screening, treatment selection, and disease monitoring than traditional tissue biopsies. In recent years, liquid biopsy has evolved as a tool for monitoring and analyzing predictive biomarkers for a variety of cancers.^31^ Liquid biopsy is a desirable approach because it is noninvasive, inexpensive, and can quickly provide physicians with information to guide decisions on treatment strategies.^32^, ^33^ Crucially, liquid biopsy can be easily repeated to follow patients during therapy, such as monitoring treatment efficacy, tumor recurrence, and mechanisms of resistance to treatment.^34^, ^35^ Liquid biopsy can dynamically and noninvasively interrogate the release of specific molecules from the tumor.

**FIGURE 1:**
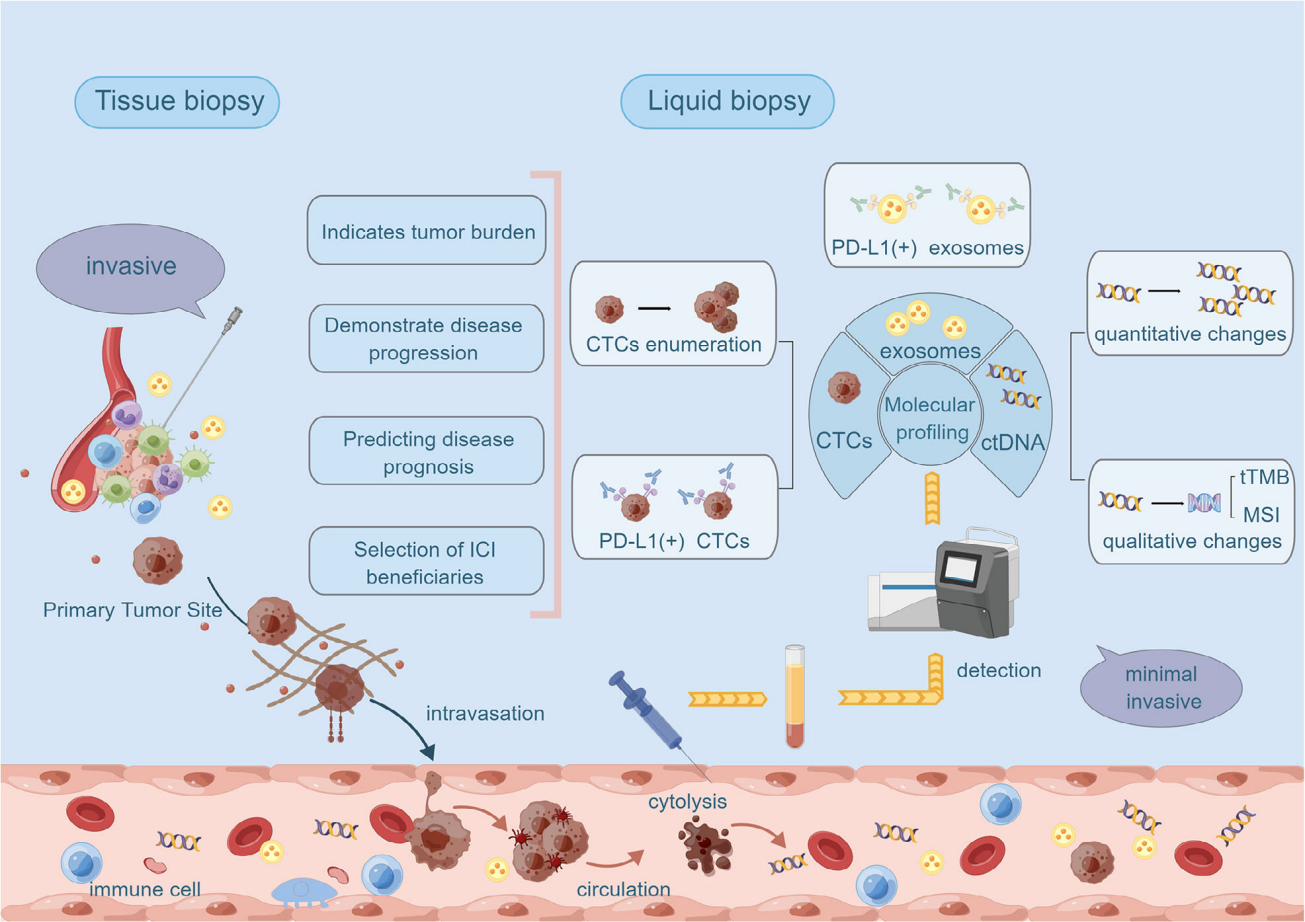
Application of liquid biopsy in immunotherapy of tumors. This schematic illustrates the advantages of liquid biopsy over tissue biopsy, such as minimally invasive and reproducible. As an essential alternative or complementary tool to tissue biopsy, liquid biopsy obtains a tumor profile by analyzing tumor‐derived components from the circulation (e.g., circulating tumor cells, circulating tumor DNA, exosomes, etc.) and can be used in critical fields, such as screening for immunotherapy beneficiaries, indicating disease progression, predicting prognosis, and so on (by Figdraw). CTCs, circulating tumor cells; ctDNA, circulating tumor DNA; ICI, immune checkpoint inhibitor; MSI, microsatellite instability; tTMB, tissue tumor mutational burden.

This review focuses on the significant role of liquid biopsy‐based techniques in extracting and analyzing tumor‐related components in blood in guiding tumor therapy. We address the limitations of the current clinical application of immunotherapy and the lack of utility of tissue biopsy, thus leading to the significant clinical value of liquid biopsy as a new testing tool. Since liquid biopsy is a new monitoring tool and the information on the contents of liquid biopsies is not widely used in clinical practice, we have collected and summarized clinical data on the current application of liquid biopsy technology in different cancers to illustrate the current development of liquid biopsy technology and the future development trend of liquid biopsy technology. The application of liquid biopsy technology in tumor therapy is a relatively new technology, and the existing review about the application of liquid biopsy in the clinic needs to be revised. We reviewed the literature published in the past several years on applying liquid biopsy components in tumor treatment. We summarized the clinical application of liquid biopsy in early diagnosis, growth monitoring, and tumor treatment. It helps to improve the knowledge of this field, promote the realization of individualized therapy, improve the efficacy of tumor therapy, and provide better therapeutic options for tumor patients (Figure 1).

## 2 LIQUID BIOPSY: DEFINITION AND METHODOLOGIES

Liquid biopsy refers to a noninvasive diagnostic or monitoring technique that involves the analysis of various biomarkers, including CTCs, cell‐free DNA (cfDNA), EVs, and other nucleic acids, proteins, or metabolites found in body fluids such as blood, urine, or CSF. Unlike traditional biopsies, which involve the surgical removal of tissue samples from a tumor, liquid biopsy allows for the detection and analysis of genetic and molecular alterations associated with diseases, such as cancer, without the need for invasive procedures.

Liquid biopsy is grounded in the understanding that tumors shed components into the bloodstream or other bodily fluids. These components carry information about the genetic mutations, epigenetic changes, and other molecular characteristics of the tumor. By analyzing these components, researchers and clinicians can gain insights into the presence of cancer, its genetic makeup, and its response to treatment. This review mainly describes the clinical applications of CTCs, ctDNA and EVs in cancer patients.

### 2.1 The detection and analysis of CTCs

CTCs are cancer cells detached from a primary tumor and entered the bloodstream, allowing them to circulate in the peripheral blood. These cells are shed from both primary and metastatic tumors and carry valuable information about the characteristics of the cancer.^36^ The detection and analysis of CTCs have become a crucial aspect of liquid biopsy techniques, offering a noninvasive means to monitor and understand cancer progression, treatment response, and metastatic potential.^37^

CTCs originate from primary tumors and metastatic sites. As tumors grow, some cancer cells acquire the ability to enter blood vessels and travel through the bloodstream. The release of CTCs into the bloodstream is a dynamic and ongoing process, and their presence can indicate an active and invasive tumor.^38^ Technologies for isolating and enumerating CTCs from blood samples have been developed. Common methods include immunomagnetic separation,^39^ microfluidic devices,^40^ and filtration systems.^41^ The ability to count CTCs provides important prognostic information, as an increased number of CTCs is often associated with a poorer prognosis.^42^ CTC analysis has clinical applications in various areas, including cancer diagnosis, prognosis, treatment selection, and monitoring. Monitoring changes in CTC counts over time can help assess treatment response, detect minimal residual disease (MRD), and identify the emergence of treatment resistance.^43^ CTCs carry genetic and molecular information from the tumor of origin. Analyzing the genomic and molecular characteristics of CTCs can provide insights into tumor heterogeneity and potential therapeutic targets. Single‐cell sequencing technologies enable the in‐depth study of individual CTCs, offering a more detailed understanding of tumor biology.^44^, ^45^

CTCs are often present in very low concentrations in the bloodstream, making their isolation and analysis challenging.^46^ The heterogeneity of CTC populations poses difficulties in capturing the full spectrum of tumor characteristics.^47^ Standardization of CTC isolation and characterization methods is an ongoing challenge in the field.^48^, ^49^

The presence of CTCs has been associated with a higher risk of metastasis and a poorer prognosis in various cancer types. CTC analysis can potentially guide treatment decisions by providing real‐time information about the evolving tumor landscape.^50^

CTCs play a significant role in advancing our understanding of cancer biology and have promising implications for personalized cancer management. As technology continues to improve, the clinical utility of CTC analysis in liquid biopsy approaches is expected to expand.^51^

### 2.2 The detection and analysis of ctDNA

ctDNA refers to fragmented DNA released into the bloodstream by cancer cells. Cancer cells undergo apoptosis (programmed cell death) or necrosis and shed small pieces of DNA into the circulation.^52^ ctDNA carries genetic information from the tumor, and its analysis is a crucial component of liquid biopsy techniques, providing noninvasive insights into the genomic landscape of cancer.^53^

CtDNA is derived from the genomic DNA of cancer cells. DNA fragments are released into the bloodstream as these cells die or undergo necrosis. Unlike normal cfDNA, ctDNA specifically originates from tumor cells and carries the genetic alterations present in the tumor. ctDNA represents a small fraction of total cfDNA circulating in the bloodstream. The proportion of ctDNA can vary depending on factors such as tumor size, stage, and biological aggressiveness.^54^ ctDNA carries genetic alterations in the tumor, including point mutations, insertions/deletions, copy number variations, and chromosomal rearrangements.^55^, ^56^, ^57^ Analyzing these genetic alterations in ctDNA can provide information about the tumor's mutational profile, heterogeneity, and potential therapeutic targets. ctDNA analysis is employed in various clinical applications, such as cancer diagnosis, prognosis, treatment selection, and monitoring. Monitoring changes in ctDNA levels over time can be used to assess treatment response, and MRD, and identify the emergence of resistance to treatment. Various techniques detect and analyze ctDNA, including polymerase chain reaction (PCR),^58^ next‐generation sequencing (NGS), digital PCR, and droplet digital PCR.^59^ High sensitivity is required for ctDNA detection due to its low abundance in the bloodstream.

**FIGURE 2:**
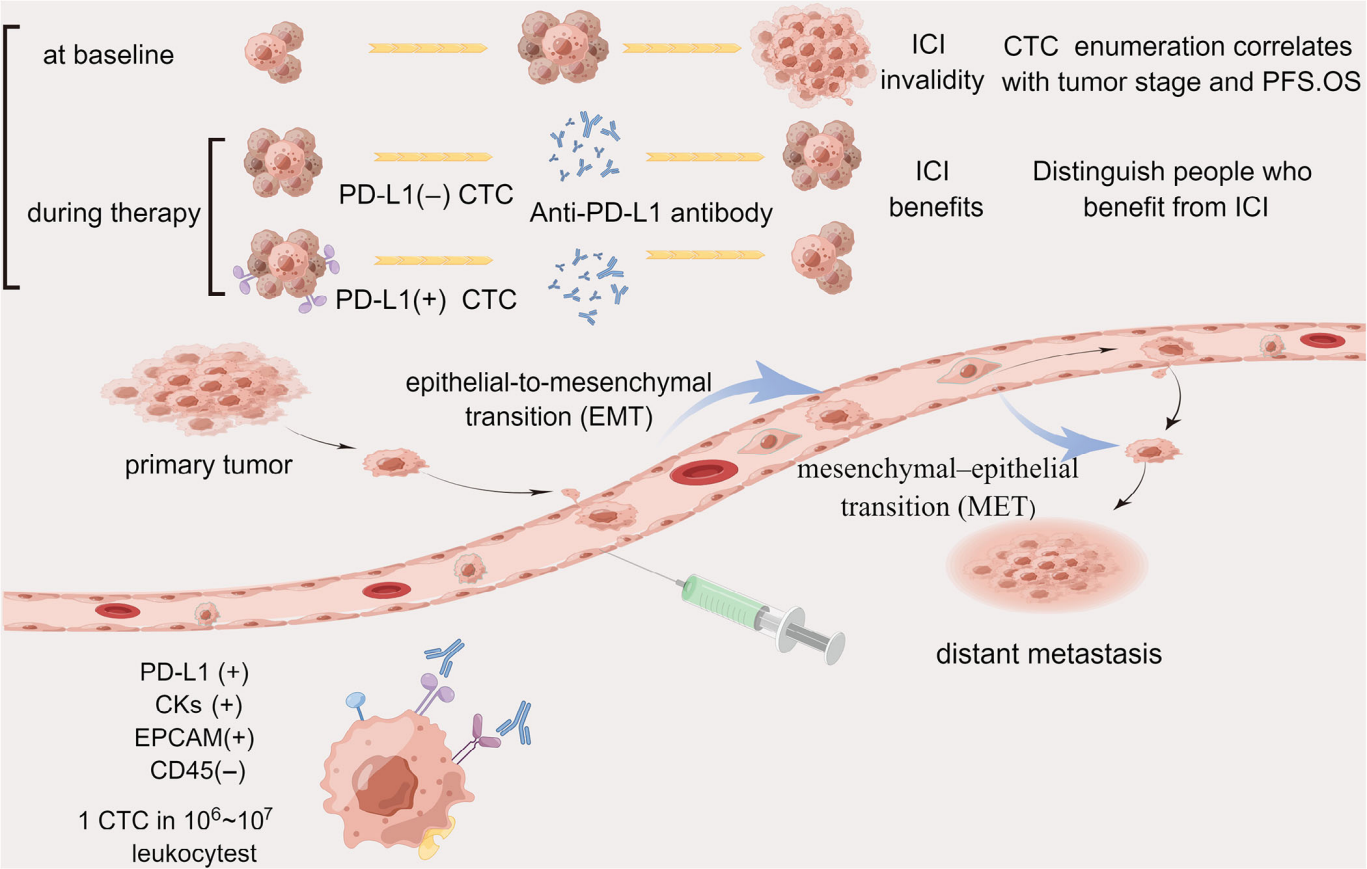
Circulating tumor cells as biomarkers in immunotherapy. CTCs are shed from the primary site of tumor and enter the circulation with blood flow for distant metastasis. CTCs are present in the blood at extremely low levels, about one per million leukocytes. They are currently analyzed and extracted mainly by the specificity of their surface molecules (e.g. PD‐L1 positive, CKs positive, EPCAM positive, CD45 negative). At baseline, the count of CTCs in blood correlates with tumor load and can be used for tumor staging and PFS, OS prediction; during the process of immunotherapy, the count and dynamics of PD‐L1(+) CTCs can be used to identify patients who benefit from ICIs treatment and also to monitor treatment response (by Figdraw). CKs, creatine kinases; EPCAM, epithelial cell adhesion molecule.

ctDNA analysis allows for assessing tumor heterogeneity, providing a snapshot of the mutational landscape across different tumor sites.^60^ Identifying subclonal mutations in ctDNA can contribute to a more comprehensive understanding of the tumor's biology. The sensitivity and specificity of ctDNA analysis methods must be carefully considered, as false positives and negatives can impact clinical decision‐making. Standardizing ctDNA analysis protocols is an ongoing challenge to ensure reliability and reproducibility across different laboratories. ctDNA analysis, particularly ctDNA analysis, has emerged as a powerful tool in cancer research and clinical practice. Its noninvasive nature and potential for real‐time monitoring make it a valuable component of liquid biopsy approaches in oncology (Figure 2).

### 2.3 The detection and analysis of EVs

EVs are small, membrane‐bound particles released by cells into the extracellular environment. These vesicles play a crucial role in intercellular communication by transporting various bioactive molecules, including proteins, nucleic acids (such as RNA and DNA), lipids, and metabolites, between cells.^61^ EVs have been implicated in various physiological and pathological processes, and their study has gained significant attention in cell biology, immunology, and cancer research.^62^ EVs are generated through budding and fission from the plasma membrane or the endosomal system. The two main types of EVs are exosomes and microvesicles (also known as exosomes or shedding vesicles), which differ in their biogenesis pathways. Exosomes are small (30–150 nm) vesicles of endocytic origin released by cells when multivesicular bodies fuse with the plasma membrane. Exosomes contain a variety of molecules, including proteins, lipids, and nucleic acids, and they can be taken up by recipient cells.^63^ Macrovesicles are larger (100–1000 nm) vesicles shed directly from the cell surface. Unlike exosomes, which originate from intracellular compartments, microvesicles are formed by outward budding of the plasma membrane.^64^

EVs carry a cargo of bioactive molecules, reflecting the composition of their cell of origin. This cargo includes proteins, nucleic acids (mRNA, miRNA, and DNA fragments), lipids, and metabolites.^65^ EVs play a key role in cell‐to‐cell communication by delivering their cargo to target cells, influencing various physiological and pathological processes, such as immune responses, tissue repair, and tumor progression. Techniques for isolating and characterizing EVs include ultracentrifugation, density gradient separation, size exclusion chromatography, and immune‐affinity capture methods. Characterization often involves assessing EVs’ size, morphology, and surface markers using techniques such as electron microscopy, nanoparticle tracking analysis, and flow cytometry.^66^, ^67^

EVs have potential clinical applications as diagnostic and prognostic biomarkers in various diseases, including cancer, neurodegenerative disorders, and cardiovascular diseases. They are also being explored for therapeutic purposes, such as drug delivery vehicles and immune modulation. EVs play a significant role in cancer progression by facilitating communication between cancer cells and their microenvironment. They contribute to tumor growth, angiogenesis, immune evasion, and metastasis. The analysis of EVs in cancer patients may provide valuable information about the tumor's molecular profile and interactions with the surrounding tissues.^68^ The study of EVs has opened new avenues for understanding cell communication and has significant implications for basic research and clinical applications. Researchers continue to explore the diverse functions of EVs and their potential as diagnostic and therapeutic tools in various diseases.^69^

## 3 LIQUID BIOPSY FOR CANCER SCREENING

### 3.1 Limitations of tissue biopsy

Early detection of tumor progression and early screening of effective treatment populations are fundamental determinants of improved overall survival (OS). However, effective biomarkers associated with the efficacy of immunotherapy drug therapy are an urgent clinical need that needs to be resolved.^70^ Currently, immunohistochemistry (IHC) on tumor tissue sections to assess PD‐L1 expression as a predictive biomarker for companion immunotherapy diagnosis is permitted for clinical application9. However, as the current gold standard for direct access to tumor profiles, there are still numerous challenges in using tissue biopsy to assess tumor status.^71^, ^72^ First, tissue biopsy‐based tumor information does not represent the complete tumor picture due to intra‐tumor heterogeneity and sampling bias. This disadvantage is commonly seen in using PD‐L1 IHC as a predictive biomarker. Second, the molecular profile of tumors evolves dynamically over time, and traditional tissue biopsies do not allow for repeat samples to be obtained in real time. Third, the therapeutic pressure exerted on tumor cells, such as targeted drugs and chemotherapeutic agents, can dynamically alter the genomic structure of the tumor. In addition to these few factors, there are still fatal drawbacks to predicting prognosis in patients using ICIs. For example, predicted treatment response based on PD‐L1 IHC and tumor mutation burden (TMB) assessment is not always definitive because of the lack of useable tissue and the low percentage of tumor cells in affected samples; despite some patients having high TMB and PD‐L1‐positive cells, immunotherapy may also fail. This phenomenon reveals the complexity of tumor immunopathology and our incomplete comprehension of immunopathology.^73^ Therefore, the status of PD‐L1 cannot be fully represented by tissue biopsy, and the bias in tissue sampling leads to the inability to receive immunotherapy. In addition, a traditional biopsy is an invasive operation that requires a probe to penetrate deep into the cancerous tissue to extract and analyze the relevant molecular structure, which has the most significant disadvantage of causing the spread of tumor cells. At the same time, many cancer patients do not tolerate repeated puncture biopsies.^74^ Therefore, tissue biopsy no longer satisfies the clinical need for tumor trait detection.^75^, ^76^

### 3.2 Advantages of liquid biopsy in cancer screening

Cancer screening using liquid biopsy is a promising and evolving approach involving analyzing various body fluids components, such as blood, to identify cancer‐associated biomarkers.^77^ Liquid biopsy offers a noninvasive and potentially more accessible method than traditional tissue biopsies. The aim is to detect cancer at an earlier, more treatable stage, improving patient outcomes and potentially reducing the need for invasive procedures. Early‐stage cancers are typically difficult to detect using traditional imaging methods.^78^ Still, a liquid biopsy can detect a small amount of DNA or tumor cells shed by tumors, improving the detection rate of early‐stage cancers. What is more, liquid biopsy is not limited to specific types of cancer; it has broad applicability.^79^ Circulating tumor markers released by different types of tumors in body fluids have unique characteristics, so liquid biopsy can be used to screen for multiple types of cancer, including but not limited to lung cancer, breast cancer, colorectal cancer (CRC), and so on.^80^, ^81^, ^82^ At the same time, for individuals known to be at high risk, liquid biopsy can be used as a regular screening method to help detect potential tumors early. This includes people with a family history, people who have received radiotherapy, and so on. A liquid biopsy allows for continuous monitoring of circulating tumor marker levels, enabling real‐time tracking of dynamic changes in tumors. This dynamic monitoring capability makes liquid biopsy suitable for one‐time screening and long‐term monitoring to detect tumor growth trends and mutations.^83^ Monitoring time has now been greatly reduced by the latest flow cytometry method, which is capable of detecting “latent” CTCs from 1 mL of blood in less than an hour (“latent” because there is no molecular evidence of malignancy at this stage), and with a high degree of sensitivity.^84^ This is undoubtedly a technological step allowing faster application of CTCs in clinical practice. This is discussed in more detail below.

### 3.3 Application of liquid biopsy in screening different types of cancers

Available scientific data indicate the importance of CTCs at different stages of CRC. Even if the current gold standard for CRC screening and diagnosis remains colonoscopy and tissue biopsy,^85^ CTC analysis may benefit better patient compliance and reduce financial burden. In this regard, a prospective clinical study was presented at ASCO GI 2018.^86^ The study was conducted on 620 patients, including 138 healthy individuals and 438 with precancerous and cancerous lesions (adenomas, polyps, and CRC stages I–IV). After processing blood samples from all 620 patients, CTCs were successfully captured and counted. The results showed an overall accuracy of 88% for precancerous and cancerous lesions at all stages of cancer disease. Further research into the clinical use of liquid biopsy as a CRC screening test has identified circulating endothelial cell clusters originating from tumors (ECC). ECC is described as benign cells created in the tumor vasculature. By identifying and counting ECC, healthy individuals can be distinguished from those with early‐stage CRC. Once specificity limitations are overcome, CTC testing may become the new gold standard for CRC diagnosis.^87^

Although CTC has obvious clinical advantages, its lack of sensitivity is also prominent. For example, at the beginning of the study, a CTC threshold of 2 per 7.5 mL of blood was set in gastrointestinal tumors. Of 102 people with a CTC level of 2 per 7.5 mL of blood, 99 (97.1%) had gastric cancer. Among 45 individuals with CTC < 2 per 7.5 mL blood, 28 (62.2%) were healthy controls. Thus, the sensitivity and specificity to distinguish patients with gastric cancer from healthy controls were 85.3 and 90.3%, respectively.^88^ The sensitivity of using CTCs to detect patients with advanced GC is higher than that of detecting patients with early GC; however, due to the low detection sensitivity, it is not recommended that CTCs cannot be used alone for GC screening in clinical practice. With the in‐depth study of CTC and the improvement of monitoring technology, CTC has a great prospect for clinical application as a screening indicator.^89^

In assessing the potential clinical value of plasma ctDNA levels as a diagnostic tool for early‐stage lung cancer, the researchers found that NSCLC patients had significantly higher plasma ctDNA levels than subjects with chronic respiratory inflammation and healthy individuals.^90^ Meanwhile, the sensitivity and specificity of ctDNA levels in distinguishing NSCLC patients from healthy individuals were 90 and 80.5%, respectively.^91^ In addition, in another study, ctDNA was detected in 100% of plasma samples from stage II–IV NSCLC patients and 50% of plasma samples from stage I NSCLC patients, with a specificity of 96% fragmentation of the mutant allele reduced to 0.02%. It was also found that ctDNA levels were highly correlated with tumor volume and could differentiate between residual lesions and treatment‐related imaging changes. This suggests that measurement of ctDNA levels may allow earlier efficacy assessment than imaging methods.^92^

The value of ctDNA methylation in early CRC screening has also been affirmed. The tumor suppressor gene septin‐9 (SEPT9) is one of the most widely studied methylation marks in the pathogenesis of CRC. One study evaluated the efficiency of the SEPT9 methylated DNA assay in diagnosing early CRC cases.^93^ The trial results showed good accuracy, leading to US FDA approval of the EpiProcolon assay as a CRC cancer screening test designed to detect SEPT9 gene methylation in DNA fragments (circulating DNA or ctDNA) released by cancer cells.^94^ The diagnostic value of this test has subsequently been extensively examined in numerous meta‐analyses.^95^, ^96^ In addition, more ctDNA methylation markers showed significant accuracy in identifying malignant tumors and precancerous lesions.^97^ A recently published study demonstrates results obtained by implementing an 11‐biomarker model based on cfDNA methylation suitable for detecting advanced adenomas and early CRC. The model achieved high sensitivity for stage I CRC and advanced adenoma and demonstrated strong specificity values in the validation cohort.^98^

In a case–control study based on breast cancer‐associated CTCs (BrAT‐CTCs), CTC enrichment was performed by multiple fluorescent immunocytochemical analyses of GCDFP15, GATA3, EpCAM, PanCK, and CD45. In prospective clinical studies in women with suspected BrC (*N* = 141), the ability of the test to distinguish cases of BrC from benign breast disease was evaluated. Results showed 100% specificity and 92.07% overall sensitivity in this case–control study, regardless of age, race, disease stage, grade, or hormone receptor status. This greatly highlights the potential of CTCs in the early screening of tumors.^99^

## 4 LIQUID BIOPSY FOR CANCER MONITORING

### 4.1 Tumor progression monitoring

The number of CTCs has been validated in patients with various tumors to be responsive to tumor burden and to reflect tumor progression. A study by Konczalla et al.^100^ used the CellSearch system to enumerate CTCs in 76 patients with preoperatively nonmetastatic staged esophageal cancer. Fifteen of 76 patients (19.7%) carried CTCs. OS and recurrence‐free survival were significantly shorter in patients with CTCs (*p* = 0.038 and *p* = 0.004, respectively). Multivariate analysis indicated that CTC status was an independent predictor of overall and relapse‐free survival (*p* = 0.007 and *p* < 0.001, respectively).^100^ This property is also present in colorectal tumors. In a prospective cohort of 149 patients with CRC who were enrolled, an analysis comparing the number of CTCs and clinicopathological characteristics of colon and rectal cancer showed a positive correlation between the number of CTCs increasing with tumor stage progression, a statistically significant phenomenon. This confirms that CTCs can reflect tumor load and that monitoring CTCs may be valuable in assessing clinical staging and estimating clinical prognosis.^101^ Chalfin et al.^102^ analyzed 183 samples from 81 patients with metastatic genitourinary cancers. They showed that having CTCs at baseline was associated with shorter OS in 75% of patients (*p* = 0.022), but CTCs did not correlate significantly with treatment response. Also, among the five morphological subtypes detected by CTCs, the presence of two specific subtypes with unique cellular characteristics at baseline and treatment was associated with poorer OS (0.9–2.3 vs. 28.2 mos, *p* < 0.0001–0.013). This study suggests that shorter survival may be related to the presence of specific CTC morphological subtypes. Future studies are needed to validate CTC heterogeneity and to test the prognostic utility of specific CTC morphologies.^102^ In studies that use circulating markers to monitor tumor progression, the persistence of CTCs is an independent predictor of tumor progression and is associated with poorer progression‐free survival and OS, which is associated with both CTC count and a specific subtype. There is no doubt that this provides an additional tool for predicting tumor progression. Many clinical studies have demonstrated the correlation between ctDNA and the prognosis of oncology patients. In a study by Miller et al.^103^ on glioma, tumor‐derived DNA in the CSF was associated with several imaging presentations, including tumor progression, tumor load, and tumor spread into the ventricular system or subarachnoid space. Tumor DNA in the CSF was associated with shorter survival following CSF collection.^103^ This suggests that analysis of the amount of ctDNA can provide relevant information on tumor progression^104^ (Table 1).

**TABLE 1:** Circulating tumor cell enumeration as a predictive biomarker.

Years	CTCs(+)	Number of subjects/cancer type	Main results	References
2019	33%	104 patients with NSCLC	CTC is present in one‐third of patients with advanced NSCLC (positivity rate 32%, 33 out of 104), and its presence is an independent predictor of lack of durable response to immunotherapy and is associated with worse progression‐free survival and overall survival.	^104^
2020	64%	47 patients with refractory colorectal cancer	Baseline assessment of CTCs may help refine patient prognosis and guide management decisions (CTCs from 30 out of 47 patients could be included in the assessment, with 10 out of 30 showing that high CTC counts predicted worse OS), but early changes in CTCs (within 2 weeks) do not provide predictive information.	^105^
2021	19.7%	76 patients with preoperatively nonmetastatic staged esophageal cancer	Fifteen of 76 patients (19.7%) harbored CTCs. It was confirmed by prospective studies that patients with CTCs showed significantly shorter overall and relapse‐free survival in the early disease stage of esophageal cancer (*p* = 0.038 and *p* = 0.004, respectively) and that CTC status is an independent, suitable, and easily used prognostic marker for clinical staging of esophageal cancer patients.	^100^
2021	48.3%	149 patients with colorectal cancer	The results of analysis comparing CTC counts and clinical pathological features in colon and rectal cancer indicated that with increased tumor stage, the number of CTCs also increased, with significant statistical differences. CTC counts in patients with colon and rectal cancer showed positive correlations with TNM staging (*p* = 0.001, 0.013, respectively), T staging (*p* = 0.021, 0.001), N staging (*p* = 0.014, 0.035), and M staging (*p* = 0.018, 0.203). Detection of serum biomarkers in CTC‐positive and CTC‐negative groups indicated a significantly increasing expression in the CTC‐positive group.	^101^
2021	75%	81 metastatic genitourinary cancer patients	In patients with metastatic genitourinary cancer, CTCs were found in 75% of patients and CTCs burden was associated with shorter OS at baseline (*p* = 0.022). Shorter survival may be associated with presence of specific CTC morphologic subtypes, PD‐L1+ CTCs, and low CD4/8 T cells in metastatic genitourinary cancer patients. A future study is warranted to validate the prognostic utility of CTC heterogeneity and detection of specific CTC morphologies.	^102^
2022	/	104 patients with metastatic renal cell carcinoma	Patients with CTC count trajectories in the highest quartile (>0.12 CTCs/mL annually) had shorter overall survival (median 17.0 months versus 21.1 months, *p* < 0.001). The prognostic importance of CTC counts was identified in this first large longitudinal study of metastatic renal cell carcinoma CTCs to date.	^106^
2024	/	189 patients with advanced breast cancer	If CTCs ≥ 5 /7.5 mL, chemotherapy should be performed. If the CTC count was low, endocrine therapy was performed, and chemotherapy was superior to endocrine therapy in 189 clinician‐recommended endocrine therapy patients (hazard ratio for death was 0.53; 95% CI, 0.36–0.78; *p* = 0.001). The CTC count can be used as a reference for selecting treatment options	^107^

Abbreviations: CTCs, circulating tumor cells; NSCLC, non‐small cell lung cancer; OS, overall survival; PD‐L1, programmed cell death‐ligand 1.

### 4.2 Tumor recurrence monitoring

In predicting tumor recurrence, a strong association was found between CTC count and recurrence in breast cancer patients, suggesting that CTC release and survival are critical steps in metastasis development.^53^ Indeed, studies involving thousands of breast cancer patients published in the last five years suggest that CTC counts before neoadjuvant therapy and at the time of initial surgery before adjuvant treatment are of great value in predicting the risk of disease recurrence.^108^, ^109^

A significant percentage of patients with early‐stage triple‐negative breast cancer (TNBC) receive neoadjuvant chemotherapy. Postoperative sequencing of ctDNA and counting of CTCs can detect microscopic residual disease and assess which patients are likely to experience disease recurrence. It is extremely important to explore the relationship between ctDNA and CTC expression and tumor recurrence and clinical regression after neoadjuvant chemotherapy in patients with early TNBC. In a phase 2 multicenter randomized clinical trial, early TNBC patients with residual disease after neoadjuvant chemotherapy were randomly assigned to receive neoadjuvant genome‐directed therapy versus physician‐selected therapy. At 24 months, distant disease‐free survival (DDFS) probability was 52% for ctDNA‐positive and CTC‐positive patients versus 89% for ctDNA‐negative and CTC‐negative patients. The combination of ctDNA and CTC provided additional information that increased sensitivity and discrimination. ctDNA‐positive and CTC‐positive patients had significantly lower DDFS than ctDNA‐negative and CTC‐negative patients. In this randomized clinical trial, detecting ctDNA and CTC in patients with early TNBC after neoadjuvant chemotherapy was independently associated with disease recurrence, an important stratification factor for future neoadjuvant trials.^110^

A multicenter phase II clinical trial of TNBC using ctDNA mutation tracking to detect molecular remnants in patients with intermediate‐ and high‐risk early‐stage TNBC evaluated the utility of prospective ctDNA surveillance in TNBC and the activity of pembrolizumab in patients with detectable ctDNA [ctDNA‐positive (ctDNA+)]. Comprehensive prospective ctDNA surveillance by digital PCR was performed to enroll patients with early‐stage TNBC and residual disease after neoadjuvant chemotherapy or phase II/III adjuvant chemotherapy. ctDNA surveillance consisted of blood sampling from three to twelve months. Of the two‐hundred‐eight patients, 185 had tumor sequencing, 171 (92.4%) had traceable mutations, and 161 entered ctDNA surveillance. The rate of ctDNA detection by 12 months was 27.3% (44 out of 161, 95% confidence interval 20.6–34.9%). Seven patients relapsed without prior detection of ctDNA. This trial is the first prospective study to assess whether ctDNA testing has clinical utility in TNBC‐directed therapy. Patients had a high rate of metastatic disease at the time of ctDNA testing. The findings have implications for future trial design and emphasize the importance of starting ctDNA testing early, that is, more sensitive and/or more frequent ctDNA testing regimes.^111^

Similar to its application in breast cancer, adjuvant chemotherapy in patients with stage III colon cancer prevents recurrence by eliminating MRD. However, it is not possible to determine which patients remain at higher risk of recurrence after completing standard adjuvant therapy. Postsurgical ctDNA analysis can detect MRD associated with CRC recurrence. In a prospective study of CRC, ctDNA was detected in 20 of 96 postsurgical samples and was associated with inferior recurrence‐free survival (hazard ratio, 3.8; 95% CI, 2.4–21.0; *p* < 0.01). ctDNA was detectable in 15 of 88 postchemotherapy samples. The estimated 3‐year recurrence‐free interval (RFI) was 30% when ctDNA was detectable after chemotherapy and 77% when ctDNA was undetectable (hazard ratio, 6.8; 95% CI, 11.0–157.0; *p* < 0.01). After adjustment for known clinicopathological risk factors, postsurgical ctDNA status remained independently associated with RFI (HR, 7.5; 95% CI, 3.5–16.1; *p* < .001). The results suggest that postsurgical ctDNA analysis is a promising prognostic marker for stage III colon cancer. Postchemotherapy ctDNA analysis can define a subset of patients at high risk of relapse despite completion of standard adjuvant therapy. This high‐risk population presents a unique opportunity to explore alternative therapeutic approaches.^112^

As the embodiment of tumor growth and metastasis, CTCs and ctDNA provide important valuable information for tumor monitoring.^113^ A more comprehensive understanding of tumor growth is a key part of cancer treatment.

## 5 LIQUID BIOPSY FOR CANCER TREATMENT

### 5.1 Liquid biopsy‐based personalized medicine and targeted therapy

The relevance of PD‐L1 expression on CTCs to predicting prognosis, stratifying patients receiving immunotherapy, and tracking treatment response is also a current research focus (Table 2). The expression of PD‐L1 on cells is considered one of the critical mechanisms by which tumors escape from immune surveillance. The survival of patients with PD‐L1(+) CTCs is lower than that of patients with PD‐L1‐negative CTCs or no CTCs has been clarified and readily accepted.^114^ The value of expression of PD‐L1 on CTCs to guide immunotherapy still needs to be supported by a large amount of research data. No uniform conclusion has been drawn to support the idea that PD‐L1 expression in CTC predicts prognosis. Still, a meta‐analysis showed that PD‐L1 expression in cancer patients' CTC significantly correlates with poor prognosis. This is the first meta‐analysis to elucidate that PD‐L1 expression on baseline affects the prognosis of cancer patients.^115^ Data from research on patients with various stages of hepatocellular carcinoma demonstrated that PD‐L1+CTCs could distinguish between early and locally advanced/metastatic HCC with accuracy. The study used a multimarker antibody‐based CTC capture platform unique for HCC CTCs to analyze PD‐L1 expression. In contrast, the OS of patients with PD‐L1 +CTCs was considerably poorer than those without PD‐L1+CTCs. Only one of five nonresponders, who worsened within four months of starting treatment, had PD‐L1+ CTCs at baseline in the subset of 10 HCC patients treated with PD‐1 blocking, as opposed to all five responders. In this controlled prospective investigation, PD‐L1+ CTCs were found to be a reliable indicator of OS. In HCC patients undergoing anti‐PD‐1 therapy, there was a significant association between the existence of PD‐L1+ CTCs and improved therapeutic response.^116^ In data from the treatment of metastatic melanoma with pembrolizumab, the availability of PD‐L1+CTC as an independent biomarker for predicting PFS was similarly shown.^117^ PD‐L1 expression on CTCs during Nivolumab treatment may predict long‐term efficacy in a longitudinal dimension over time, according to a different article on the longitudinal evaluation of Nivolumab for NSCLC. This study followed 45 patients for a long time and compared PD‐L1 expression on CTCs before treatment with it at 4 weeks, 8 weeks, 12 weeks, and 24 weeks or until progressive disease.^118^ Similarly, changes in the proportion of PD‐L1‐positive CTCs were associated with disease outcomes in urothelial carcinoma (UC). Nine of the 11 patients who reached disease control (DC) had significantly lower PD‐L1‐positive CTC counts (*p* = 0.01). In four patients with progressive disease, this was higher or unchanged. Thus, this study confirms that PD‐L1‐positive CTCs can be used as real‐time molecular biomarkers for personalized immunotherapy and that sustained changes in PD‐L1‐positive CTCs respond to the effects of PD‐L1 blockade therapy. However, the number of patients included was small, and the number of study subjects still needs to be expanded to give more reliable conclusions.^119^ In another study on treating advanced UC with immunotherapy, an increase in the percentage of PD‐L1‐positive CTC was strongly associated with disease progression.^120^ Opposite results were found in the NSCLS population treated with ICI, where the presence of PD‐L1+CTC at baseline did not correlate significantly with the outcome, a higher baseline PD‐L1+CTC count (≥1%) was observed in the nonresponder group (PFS < 6 months) (*p* = 0.04), and PD‐L1+CTC was observed in all progressing patients. This phenomenon may predict the development of drug resistance.^121^

**TABLE 2:** Clinical application of PD‐L1(+) CTCs as biomarkers in immunotherapy.

Years	PD‐L1(+) CTCs/ CTCs	Number of subjects	Main results	References
2018	74%	35 patients with different advanced gastrointestinal tumors	Revealing the abundance of CTC at high PD‐L1 concentrations prior to treatment can be a predictor for screening patients for immune checkpoint blockade therapy, and dynamic changes in CTC can indicate treatment response at an early stage.	^126^
2018	83%	96 patients with NSCLC	PD‐L1 positivity was higher in CTCs than in tissues and a higher baseline PD‐L1+CTC count was observed in people not responding to ICI treatment.	^121^
2019	64%	Metastatic melanoma receiving pembrolizumab	Longitudinal changes in PD‐L1(+) CTC from pre‐treatment to disease progression are instructive in assessing treatment efficacy.	^117^
2020	63%	49 patients with advanced UC	Progression of disease in immunotherapy may be associated with an increase in the percentage of PDL1‐positive CTC.	^120^
2021	65%	23 patients with urothelial carcinoma	The number of PD‐L1‐positive CTCs was significantly decreased in those who achieved disease control.	^119^
2021	82%	45 patients with advanced NSCLC	Evaluation of longitudinal changes in the CTC status from prior to treatment to disease progression is informative for the assessment of clinically relevant information. The prognosis of patients treated with nivolumab may be predictable by evaluating the PD‐L1 expression on CTCs at week 8 and comparing with that at the baseline.	^118^
2021	9.4%	54 patients with advanced NSCLC	PD‐L1 expression concordance between tumor tissues and CTCs was low (54%). A significant correlation was found between the presence of CTCs and PD‐L1‐positive CTCs and poorer overall survival, but PD‐L1 expression in tumor tissues was not.	^114^
2021	82.2%	155 patients with various advanced cancers	The number of PD‐L1‐high CTCs before treatment and their dynamics during treatment are proportional to the treatment effect.	^127^
2022	/	47 HCC patients receiving the triple therapy	The results showed that patients who received triple therapy with fewer PD‐L1+ CTCs at baseline had higher objective response rates (ORRs) and overall survival (OS).	^123^
2022	/	82 patients with pathologically confirmed NSCLC	This is a single‐blind prospective study evaluating changes in PD‐L1 expression in tumor‐associated cells in blood samples before (T0) and after (T1) treatment with ICI (ICI, 41 individuals) or without ICI (no ICI, 41 individuals). Among patients treated with ICI, those with increased PD‐L1 expression between T0 and T1 had significantly better PFS (HR, 3.49; 95% CI, 1.5–8.3; *p* = 0.0091) and OS (HR, 3.058; 95% CI, 1.2–7.9; *p* = 0.0410).	^128^
2023	53%	49 patients with NSCLC	The detection rate of PD‐L1 expression was higher in CTC than in tumor tissues (53.0 versus 42.1%). There was no correlation between the two. Patients with PD‐L1 expression on tissue or CTCs had a median progression‐free survival (mPFS) of 5.6 months (*n* = 36, 95% confidence interval [CI] 3.6–7.5 months), significantly longer than those without PD‐L1 detection (*n* = 9, mPFS of 1.4 months, 95% CI 1.3–1.5 months, log‐rank *p* = 0.032).	^129^

Abbreviations: HCC, hepatocellular carcinoma; HR, hazard ratio; ICI, immune checkpoint inhibitors; PFS, progression‐free survival; UC, urothelial carcinoma.

All of the above data indicate that PD‐L1 levels on CTC have promising clinical utility as predictive biomarkers for screening effective patients for immune checkpoint blocker therapy in the population before treatment and that dynamic changes in PD‐L1 expression on CTC during treatment are valuable for assessing and monitoring response to therapeutic aspects.^122^

In contrast, a study enrolled 47 HCC patients who received triple therapy (ICI combined with intensity‐modulated radiotherapy and antiangiogenic treatment). The results showed that patients with fewer PD‐L1+ CTCs at baseline had higher objective response rates (ORRs) and OS. A cutoff of 2 PD‐L1+ CTCs was found to have the highest predictive power for ORRs in this trial. This is not the same as the predictive effect of ICIs alone, and more studies are needed to explore the impact of combination therapy on PD‐L1+ CTCs.^123^ In addition, changes in PD‐L1 in CTCs can predict the treatment modalities except for immunotherapy. Increased PD‐L1/PS6 expression in CTCs of NSCLC patients treated with Osimertinib suggests activation of the corresponding pathway, which may be associated with poor clinical outcomes.^124^ Statistics from the last two years show that the value of PD‐L1 expression on CTC in predicting prognosis before treatment and its dynamic changes in response to treatment is prospective in immunotherapy. However, predicting treatment response when combined with other therapies is questionable. A large amount of data is still needed to support the utility of PD‐L1 expression on CTCs in various cancer types. Realizing its quantitative detection and related threshold determination is essential for future development.^125^

### 5.2 Liquid biopsy‐based prediction of treatment response and resistance

MSI is a molecular tumor phenotype caused by mismatch repair (MMR) system defects, which limits the correction of spontaneous mutations in short repetitive DNA sequences named microsatellites.^130^ MSI and MMR gene alterations, which are found in tumor tissue, have been utilized as biomarkers to predict ICI effectiveness.^131^ However, tumor biopsy IHC is invasive, thus does not allow for long‐term monitoring of immunotherapy efficacy. Therefore, many studies have tried to prove that detecting MSI/MMR from ctDNA can be used as a substitute diagnosis for tissue biopsy. Chakrabarti et al.^132^ investigated patients with pancreatic ductal adenocarcinoma and found MSI detected in blood with Guardant360 could identify tumor tissue MSI‐H patients and even determined one more MSI‐H patient than IHC. Consistency was also demonstrated in patients with metastatic prostate and pancreatic cancer.^133^, ^134^ However, the results are unreliable due to insufficient data samples, and a large amount of data is still needed to verify the consistency of MSI in liquid biopsies and tissue biopsies. According to Saeed et al., the ability to test MSI noninvasively with ctDNA enabled the early identification of responders from nonresponders.^135^ That is to say. We suggest that the presence of MSI‐H in a liquid biopsy test predicts a solid response to ICI. In addition to the baseline level of MSI status to predict immunotherapy efficacy, the varying level of MSI allelic frequencies during immunotherapy may reflect the response,^136^ which can be used to monitor the effects of treatment dynamically. Therefore, increasing studies have started detecting MSI mutations at different times to explore the pattern of MSI changes during treatment. Silveira developed ddPCR assays targeting microsatellite markers before and during therapy and showed their dynamic during treatment correlated well with clinical response, which demonstrated undetectably or dramatically decreased ctDNA concentrations (>90% MAF reduced) over the first weeks of therapy of patients who were responsive to pembrolizumab.^136^ In two prostate cancer patients who underwent ctDNA testing before and throughout immunotherapy, Duchemann et al. found that one had completely cleared all somatic variations. The other had significantly reduced somatic variants.^131^ We cannot only compare MSI status increased with baseline levels but also predict the effect of immunotherapy by detecting residual MSI changes throughout treatment. Georgiadis conducted a study where they analyzed cfDNA in patients with metastatic cancers who were undergoing pembrolizumab therapy. They found a negative relationship between overall and progression‐free survival and the levels of residual MSI allele in cfDNA at the last dose. This suggests that the residual MSI allele in cfDNA could be a predictive marker for treatment response earlier than radiographic imaging.^137^

Research reveals that exosomal PD‐L1 levels rise over time in some patients receiving immunotherapy who often show no response or continued disease progression.^138^, ^139^ In conclusion, high levels of exosomal PD‐L1, both at baseline levels and after treatment, may lead to poor efficacy. Exosomal PD‐L1 can mimic the immunosuppressive effects of tumor cell membrane PD‐L1, which has the same membrane topology and thus may firmly bind to immunosuppressants to produce drug resistance.^140^ Not only can tumor‐secreted exosomes act directly on antibody drugs to create drug resistance, but studies have also found that exosomes can alter the cells in the tumor microenvironment (TME), which can have immunosuppressive effects and interfere with the immunotherapy.^141^ In the exosomal PD‐L1‐mediated resistance pathways, Wei et al.^142^ highlight HMGB1‐induced resistance by upregulating PD‐L1+ exosomes. Overall, currently the most popularly accepted is that in drug‐resistant populations, the secretion and release of exosomal PD‐L1 are regulated through various pathways to modulate the body's immune system, thereby rendering immunosuppressive drugs ineffective.^143^, ^144^, ^145^, ^146^ Today, many studies are trying to target exosomal PD‐L1 and related modulators to dismantle the immunotherapy resistance problem.^147^, ^148^

### 5.3 Liquid biopsy‐guided treatment decisions

In a retrospective study investigating tTMB in ICI for NSCLC by Gandara et al.,^149^ the value of tTMB in immunotherapy was initially validated, with results showing a strong correlation between tTMB and bTMB, while bTMB ≥ 16 was determined to serve as a clinically significant and technically reliable predictive cut‐off point for NSCLC treated with ICI in multiple analyses. Notably, the results showed that bTMB was not associated with high PD‐L1 expression, which allowed the two to be applied in combination at different levels to improve predictive capacity, respectively, and to validate patients with high PD‐L1 expression and bTMB ≥ 16 had the best PFS outcome. This is the first demonstration that tTMB correlates with the clinical benefit of treatment with ICIs.^149^ A follow‐up study also confirmed that ICI‐treated patients with higher standardized bTMB had significantly better final clinical outcomes.^150^ NGS has shown high utilization in terms of economic and practical value as a tool to observe tumor gene mutations. Khagi et al.^151^ screened and analyzed 54−70 of these genes by NGS monitoring ctDNA samples from different cancer types treated with ICI; among all included 69 patients, based on total and variants of unknown significance (VUS) alterations to assess disease progression, the results showed statistically significant improvements in PFS and OS correlated with the levels of total alteration number and VUS, most notably the median PFS for responders and nonresponders with VUS > 3 was 23 months, 2.3 months (*p* = 0.0004). This implies an auspicious role of ctDNA as a marker to predict the outcome of ICI treatment.^151^ To improve the sensitivity of ctDNA assays, personalized gene panels interrogating multiple mutations based on raw tumor sequencing, analysis of higher volumes of plasma, and repeat sampling to improve the sensitivity of mutation detection can be specifically recommended.^152^ TMB measured by whole exome sequencing (WES) or genetic cancer panel (CGP) is associated with immunotherapy response. Due to the impracticality of WES for clinical use, effective CGP monitoring of bTMB is currently designed as a substitute option. For example, a CGP‐designated NCC‐GP150 was designed and practically demonstrated in a cohort study using the Cancer Genome Atlas database.NCC‐GP150, comprising 150 genes, showed a stable correlation with TMB estimates by WES, especially when synonymous mutations were included. In contrast, the TMB estimated by the NCC‐GP150 panel showed a higher correlation with the TMB estimated by the WES than most of the randomly sampled 150‐gene panel.^153^ These investigations strongly supported the bTMB found by the targeted NGS panels. Meanwhile, a strong panel of targeted NGS is developed, increasing the sensitivity of ctDNA assays. The efficacy of bTMB as a biomarker for liquid biopsies in predicting the therapeutic effect of ICI immunotherapy requires further clinical research. In addition to the analysis of the number of ctDNA mutations, where mutations in individual genes are also closely associated with the effects of immunotherapy, the AT‐rich interaction domain 1A (ARID1A) is considered to be related to anti‐tumor immunity. It has been shown that ARID1A alterations can diminish MMR and are related to impaired DNA damage repair. In a trial that included 71,301 patients with advanced solid tumors and analyzed their cfDNA by NGS, 5% of them had ≥1 deleterious ARID1A alteration, which is exceptionally close compared with the 6% mutation frequency in tissues, predicting the promise of ARID1A analysis in liquid biopsies as a surrogate for tissue biopsies to predict immunotherapeutic efficacy.^154^

However, some studies also found that high TMB assessed by tTMB or bTMB was not associated with better survival outcomes.^155^, ^156^ The emergence of this phenomenon does not refute the value of bTMB or tTMB in tumor immunotherapy,^157^ probably due to the current inability to define a threshold for high TMB and the fact that these studies need to be more intensive as a consequence of the different cancer types, technical issues and the limited number of subjects studied.

**FIGURE 3:**
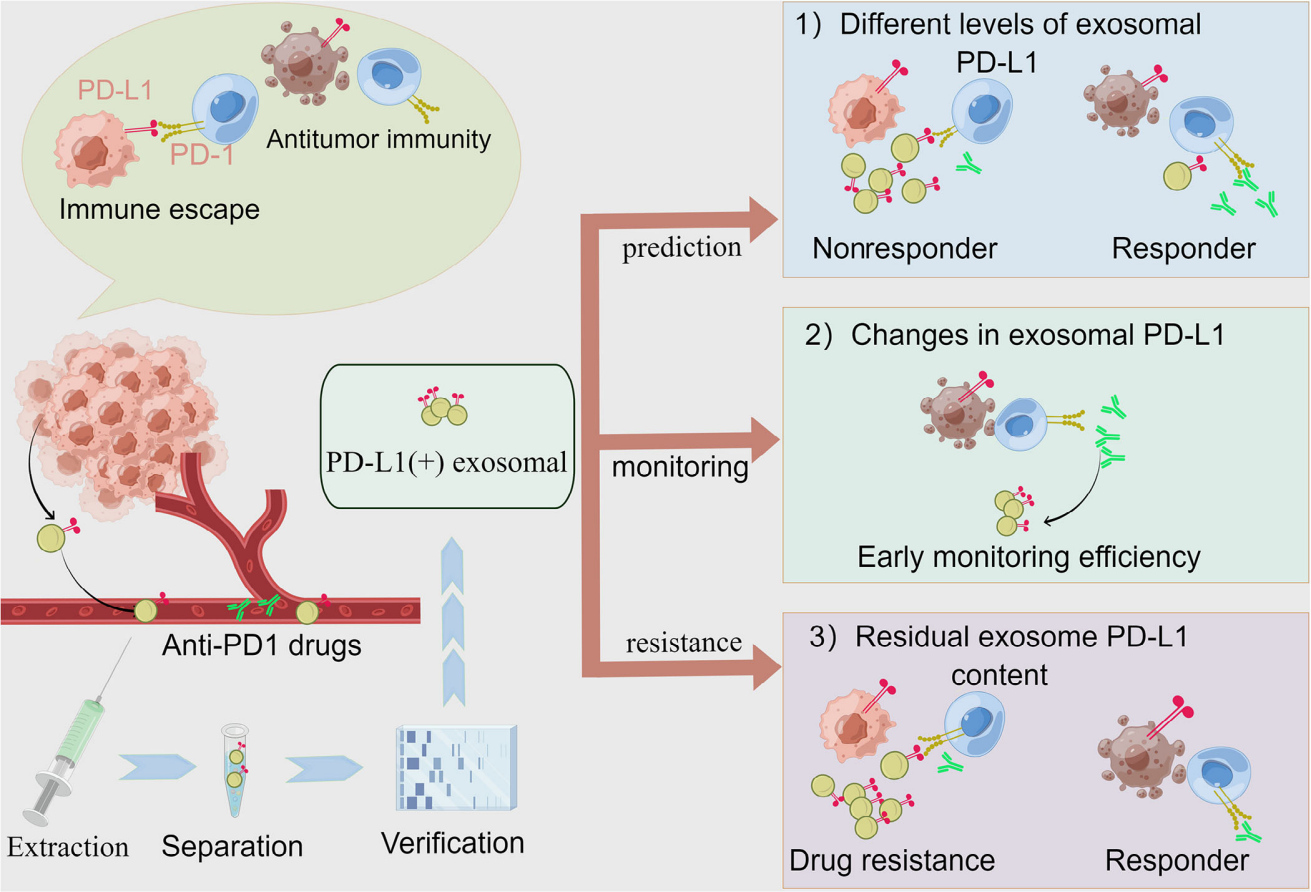
Three hints of the significance of exosomal PD‐L1 levels in immunotherapy. With the development of detection technology, exosomal PD‐L1 levels could be applied to anti‐PD‐1 therapy in three ways: (1) high levels before treatment to predict nonresponders; (2) elevated exosomal PD‐L1 early in treatment to suggest effective treatment; (3) high levels of exosomal PD‐L1 to suggest drug resistance (by Figdraw).

The temporal and spatial heterogeneity of tumors and the redistribution of PD‐L1 through exosomes result in the detection of PD‐L1 status by IHC as an instantaneous capture of local tumor tissue,^158^, ^159^ thus not fully predicting the efficacy of anti‐PD‐1/PD‐L1 immunotherapy, for which we speculate that exosomal PD‐L1 assays are superior to or complementary to IHC assays. It should be noted that exosomal PD‐L1 could not reflect the PD‐L1 IHC status, which was proved in NSCLC and breast cancer.^158^, ^160^ Exosomal PD‐L1 has been shown to attenuate the anti‐tumor capacity of immune cells, causing immune escape in breast cancer.^159^ Therefore, exosomal PD‐L1 has a different suggestive meaning in immunotherapy than the PD‐L1 IHC status. It is reported that anti‐PD1 therapy relieves the immunosuppression of PD1+CD8+ T cells and enhances IFN‐γ expression, resulting in high PD‐L1 expression in the TME, which implies that the elevation of exosomal PD‐L1, indicating the tumor cells' adaptive response to T cell resurrection, predicts the response to immunotherapy during the initial stages of treatment, which facilitates the early monitoring of immunotherapy efficacy.^161^ Interestingly, this increased PD‐L1 expression which selected the responders to immunotherapy, may not suppress immune response because PD1+T cells are entirely bound by anti‐PD‐1 antibodies.^162^ Further, significant differences in PD‐L1 abundance at different treatment times have been found for different immunotherapy profiles.^163^ We found that today's research on exosomal PD‐L1 predicting immunotherapy includes two main directions: (1) the correlation between exosomal PD‐L1 levels before treatment and immunotherapy stratification; (2) the correlation between changes in exosomal PD‐L1 and immunotherapy efficacy during treatment. In a study reported in 2018, stage III–IV melanoma patients were enrolled for treatment with pembrolizumab and blood tests before and after the treatment. Researchers collected exosomes by ultracentrifugation at 100,000×*g* for 2 h, which allowed discrimination of microvesicles; then purified exosomes using the exosome isolation kit, determined the concentration of exosomes using NanoSight NS300, and detected exosomal PD‐L1 by ELISA. They found clinical outcomes were worse when exosome PD‐L1 levels before therapy were higher and exceeded 1.03 ng/mL because T cells cannot undo the solid inhibitory effect.^164^ That is to say, high levels of exosomal PD‐L1 above a certain threshold before treatment indicate ineffective immunotherapy,^165^ where T cells are at the “exhaustion stage” and hardly be resurrected by the anti‐PD‐1 therapy. However, we need to investigate further whether lower or even undetectable levels of exosomes before treatment suggest that immunotherapy is more effective.^166^ In addition, Theodoraki et al.^167^ and Cordonnier et al.^168^ found that a reduction in exosomal PD‐L1 during treatment was related to the tumor response in the scan. In contrast, other studies have found diverse variations in exosomal PD‐L1 amount. Chen et al.^164^ found that the amount of exosomal PD‐L1 rose dramatically in responders, primarily during the first 6 weeks of treatment, while Del Re et al.^169^ found that exosomal PD‐L1 significantly decreased in responders after two months of immunotherapy, which suggests that the change in exosomal PD‐L1 levels of responders to immunotherapy would be closely related to the duration of treatment, where the broad change is first elevated and then decreased (Figure 3).

Although exosomal PD‐L1 is a promising research hotspot, there are still many pressing questions in the research process: (1) the predictive significance of exosomal PD‐L1 applies to which cancer types?; (2) the quantitative and temporal thresholds for changes in exosomal PD‐L1 content; and (3) the difficulty in standardizing the technique of exosomal PD‐L1 isolation and quantitative measurement. Meanwhile, whether exosomal PD‐L1 changes in the patients who fail immunotherapy with high PD‐L1 expression and those who are effective with low PD‐L1 expression can compensate for the blind spot of IHC detection and early prediction of people who are influential with immunotherapy needs to be investigated. Therefore, clinical studies are required to evaluate the predictive relevance of exosomal PD‐L1 and improvements to the relevant test methodologies. Besides, a new study combined four plasma EV‐derived proteins containing exosomal PD‐L1 into an EV‐score that predicts immunotherapy at baseline and dynamically prognoses patient survival while on treatment.^170^ Thus, we conjecture that taking exosomal surface membrane proteins, which may be involved in immune regulation, including exosomal PD‐L1, into account may give better predictions for immunotherapy efficiency (Table 3).

**TABLE 3:** Exosomes as a predictive biomarker for immunotherapy.

Years	Number of subjects/cancer type	Main results	References
2018	39 patients with Stage III to IV melanoma treated with pembrolizumab	Clinical outcomes were worse before therapy when exosome PD‐L1 levels were higher and exceeded 1.03 ng/ml and the level in the clinical responders rose sharply, mostly following six weeks of treatment, especially a fold change greater than 2.43 at week 3−6.	^171^
2018	18 patients with melanoma and 8 NSCLC treated with nivolumab and pembrolizumab	The amount of exosomal PD‐L1 substantially rose in those with disease progression and reduced in those responding to therapy after two months.	^169^
2019	18 patients who received a combination regimen that included immunotherapy	Tumor‐derived exosome /total exosome ratios decreasing may predict the efficiency of immunotherapy.	^168^
2020	44 patients with various advanced tumors treated with anti‐PD‐1 therapy	The nonresponders’ exosomal PD‐L1 levels were noticeably greater than those of the responders at the baseline.	^165^
2020	100 melanoma patients with anti‐PD‐1 or targeted therapies	The ΔExoPD‐L1 cut‐off is a more accurate marker than PD‐L1 expression in tumor tissues, and may be used to stratify patients into two groups with remarkably different survival and progression‐free survival.	^146^
2020	30 patients with advanced EGFR/ALK wild‐type (WT) NSCLC who received PD‐1/PD‐L1 inhibitors	The plasma exosomal miRNA(Hsa‐miR‐320d, hsa‐miR‐320c, and hsa‐miR‐320b) were identified as potential biomarkers for predicting the efficacy of immunotherapy in advanced NSCLCs.	^172^
2021	Culture system for HMGB1/RICTOR‐disrupted HCC cell lines and addition of atezolizumab to it	The noncoding regulatory role of HMGB1 in HCC upregulates PD‐L1+ exosomes, which reduces apoptotic cells’ frequency and leads to tumor progression, and may explain treatment resistance and predict immunotherapy.	^142^
2021	The mice with NSCLC	Xenograft mice with higher exosomal circUSP7 expression displayed an apparent resistance to anti‐PD1 therapy, as well as worse outcome.	^173^
2021	12 cancer types such as melanoma, urothelial cancer, and renal cancer.	High tumor‐derived exosome scores was associated with shorter overall survival, which showed TEX‐score may predict Immune checkpoint inhibitor response in melanoma.	^174^
2021	38 metastatic NSCLC patients treated with nivolumab and pembrolizumab	Levels of PD‐L1 showed an increase at 3 months in patients who progressed	^169^
2022	7 posttreated cancer patients, 3 prior‐treatment patients and 6 healthy controls	The different abundance of PD‐L1 by integrated microfluidic exosome isolation and detection system reflects differences in sensitivity toward immune response, which provide useful guides to design immunotherapy strategies for different types of tumors.	^163^
2022	33 advanced NSCLC patients for retrospective and 39 of that for prospective	An elevation of exosomal PD‐L1 after 9 ± 1 weeks appeared in patients who did not respond to immunotherapy and independently predicted worse clinical outcome.	^159^
2022	112 GC patients received ICI‐related therapies	The study combined four plasma EV‐derived proteins, which contained exosomal PD‐L1, into an EV‐score that predicts immunotherapy at baseline and dynamically prognoses patient survival while on treatment.	^170^

EGFR/ALK, epidermal growth factor receptor/ anaplastic lymphoma kinase.

## 6 CLINICAL APPLICATIONS AND FUTURE PERSPECTIVES

The advantage of liquid biopsy lies in its convenient and comprehensive approach to understanding tumor growth and changes. Currently, there is an increasing number of clinical applications for this technique.^175^ CTCs, as the most direct indicators of tumor burden and metastasis in liquid biopsies, play a crucial role in early detection and monitoring of tumor growth. The expression of PD‐L1 on the surface of CTCs is associated with tumor immunotherapy. As early as 2018, comparing the expression levels of PD‐L1 in CTCs before and during treatment can reflect the effectiveness and prognosis of treatment. Their research developed an immunofluorescence assay for PD‐L1 expression levels on CTC that can be classified into four categories: PD‐L1 negative, PD‐L1 low, PD‐L1 medium, and PD‐L1 high. The DC rate among the 35 patients receiving ICIs for advanced gastrointestinal cancers is significantly greater in PD‐L1 high patients (48%) than in the other patients (14%). This initial investigation shows a correlation between PD‐L1 measurement in CTCs and treatment responsiveness to PD‐1 inhibitory medication.^126^ Similarly, 155 patients with different advanced cancers treated with ICIs were included. CTCs were enriched and counted using the Pep@MNPs method. PD‐L1 expression levels were analyzed by immunofluorescence and assessed semi‐quantitatively in four categories (negative, low, medium, and high). After eliminating the bias between different cancer types, the results showed that: (1) before treatment, the number of PD‐L1 positive CTCs and the ratio of PD‐L1 high expressing CTCs were proportional to the treatment effect. (2) During treatment, the decrease in the number of PD‐L1 positive CTCs and the ratio of PD‐L1 high expressing CTCs suggested a beneficial response to PD‐1/PD‐L1 inhibitors. (3) Also, patients with PD‐L1 high CTCs had significantly longer PFS and OS at the prognostic level. These results demonstrate their predictive power before treatment and feedback on treatment from dynamic changes during treatment.^127^

The number of clinical studies on ctDNA has increased in recent years. Many clinical studies have demonstrated the correlation between ctDNA and the prognosis of oncology patients. In a study by Miller et al.^103^ on glioma, tumor‐derived DNA in the CSF was associated with several imaging presentations, including tumor progression, tumor load, and tumor spread into the ventricular system or subarachnoid space. Tumor DNA in the CSF was associated with shorter survival following CSF collection.^103^ This suggests that analysis of the amount of ctDNA can provide relevant information on tumor progression.

The hTERT (telomerase reverse transcriptase) qPCR method was used to quantify cfDNA levels in the study by Mondelo‐Macia et al.^176^ Before treatment of NSCLC with pabolizumab, hTERT cfDNA levels at baseline were not competent as a predictive marker of prognosis. Moreover, during the treatment of NSCLC with pabolizumab, blood samples were collected pre‐treatment, at week 6 and week 12, respectively, and disappointingly, pre‐treatment hTERT cfDNA levels did not correlate with ICI treatment showed a correlation. Still, after 12 weeks of longitudinal treatment analysis, the disease progression population could clearly be distinguished by hTERT cfDNA levels.^176^ The presence of ctDNA in patients with early‐stage cancer is associated with aggressive disease. It predicts poor clinical outcomes that have been demonstrated in numerous cancer types.

Similarly, failure to clear ctDNA during treatment reflects an increased risk of treatment resistance and metastatic recurrence, such as breast cancer.^177^, ^178^ ctDNA as a dynamic predictive marker can track response to immunotherapy in patients with MSI‐high CRC. Patients who progress during immunotherapy have significantly elevated ctDNA; patients in partial remission have decreased ctDNA, and patients in complete remission have reduced or even undetectable ctDNA.^179^ This data result is also found in NSCLS.^180^ Powles et al.^181^ evaluated the outcomes of 581 patients who underwent surgery and underwent ctDNA assessment from a randomized phase III trial of the adjuvant Atezolizumab with the observation of operable uroepithelial carcinoma. At the start of treatment, 214 (37%) patients were found to be ctDNA positive with a poor prognosis (*p* < 0.0001). During treatment, ctDNA‐positive patients had better disease‐free survival and OS in the Atezolizumab group compared with the observation group. For ctDNA‐negative patients, there was no difference in disease‐free survival or OS between treatment groups. ctDNA clearance was higher in the Atezolizumab group (18%) than in the observation group (4%) at 6 weeks (*p* = 0.0204). This research elucidates the significance of ctDNA content in immunotherapy from a comprehensive perspective.^181^ Identifying the pseudoprogression of tumors has been a challenge. In a clinical study that longitudinally analyzed ctDNA and pseudoprogression of metastatic melanoma, enrolling 125 melanoma patients who received receiving PD‐1 antibodies alone or in combination with ipilimumab, the results showed that ctDNA predicted pseudoprogression with a sensitivity of 90% (95% CI, 68−99%) and a specificity of 100% (95% CI, 60−100%).^182^ Similarly, plasma samples were sequenced using WES in studies using ICIs for melanoma. CtDNA levels correlated with increasing tumor size in longitudinal ctDNA analysis. In contrast, ctDNA levels decreased immediately after a surge in patients exhibiting pseudoprogression, suggesting the potential of ctDNA analysis in distinguishing pseudoprogression from valid progression.^183^

Liquid biopsies are primarily employed as a supplemental test to tissue biopsy and have not been deemed a standard method for verifying and diagnosing various disorders, including cancer.^184^ However, in the era of immuno‐oncology, the use of liquid biopsy is being actively investigated, and some promising results are sure to lead to rapid transfer to routine clinical practice. This review compiles data from recent studies on liquid biopsy techniques for predicting prognosis and treatment response in various cancers. The value of liquid biopsy as a whole cannot be ignored. There are still some problems to overcome. It is unclear whether liquid biopsy represents all genomic clones within a single tumor or a specific subregion of cancer. Inter‐assay heterogeneity due to the different detection platforms is also an obstacle. Torga et al.^185^ found significant differences in the concordance of genetic alterations analyzed by diverse testing platforms in the same patient. Low genetic profiling congruence could jeopardize the clinical benefit of personalized medicine.^185^ In addition, some biomarkers identified by liquid biopsy are challenging to capture.

Furthermore, isolation of plasma requires specific and sensitive methods, and standardized methods or protocols for isolating the circulating biomarkers and interpreting the results are lacking.^186^ For instance, sample collection for liquid biopsies must be standardized to ensure that molecular markers are stable and measurable.^187^ Meanwhile, the release of biological materials used for liquid biopsies (e.g., urine and blood) can be influenced by microenvironmental factors.^188^ In addition to technical challenges, obstacles must be overcome before the liquid biopsy is used in clinical. For example, we found that immunotherapy is effective even in patients with low PD1 expression or low TMB status.^189^ And few studies have found a relationship between specific changes in the levels of the markers mentioned above and the efficacy of immunotherapy. So, people started to build multi‐factor prediction models to increase prediction effectiveness and to measure the weight of single factors by machine algorithms to achieve more accurate predictions. Kong et al.^190^ found that genes with similar phenotypes are mostly colocalized in specific regions of protein–protein interaction networks, performed network‐based analysis by machine learning (ML), and predicted immunotherapy response more accurately than other conventional biomarkers. Likewise, a 24‐gene RNA signature using an ML strategy drawn the same conclusion.^191^ Therefore, we speculate about enhancing the efficacy of immune prediction by combining the conventional ICI treatment biomarkers through ML, which may analyze a variety of data to find fascinating links, offer insight, and spot trends.^192^ Reviewing the previous studies, we found that ML has been used to predict immunotherapy from a genetic or transcriptomic perspective alone or together.^171^, ^193^, ^194^, ^195^, ^196^ Still, many studies have not combined ML with the relevant substances involved in the liquid biopsy technique we mentioned earlier to predict ICI efficacy. Cheerfully, attempts have recently been made to build ML based on peripheral blood tests. A study got an 88‐gene panel via ML. They demonstrated improved prediction to immunotherapy as compared with the traditional TMB.^197^ Zaitsev et al.^198^ introduced a decision tree ML algorithm called Kassandra. This algorithm was trained on RNA profiles from blood‐sorted cells and incorporated into millions of artificial transcriptomes. The purpose of this algorithm is to accurately reconstruct the TME and enhance the predictive power of existing biomarkers for immunotherapy response.^198^ Park et al.^199^ developed the serum proteomic tests through ML and effectively stratified the cancer patients into groups with good and poor treatment outcomes with immunotherapy independent of PD‐L1 expression. Through existing studies, we can predict immunotherapy efficacy using machine algorithms to design gene panels, reconstruct TME by blood RNA analysis, and synthesize proteomics. However, many clinical studies need to support the clinical significance of the above prediction models based on machine algorithms. We advocate for more scholars to optimize liquid biopsy‐based prediction models with machine algorithms. In addition, the analysis suggests combining multiple liquid biopsy biomarkers may better predict outcomes than any single biomarker.^149^ Integration of nonliquid components, such as radiographic analysis and tumor characterization,^200^ as well as multidimensional approaches involving blood‐based proteomic testing,^201^ are also needed for combined application to improve predictive capabilities. In short, liquid biopsy is not now a part of standard immuno‐oncology therapy monitoring. With the improvement of monitoring efficiency of liquid biopsy technology and the study of deeper mechanisms of liquid biopsy‐related biomarkers, liquid biopsy can be applied as an adjunctive examination to tissue biopsy in immunotherapy of tumors and even make up for the deficiency of tissue biopsy. There is still a need for large‐scale clinical data to support clinical applications, and funding is a massive challenge due to the high price of the tests. Still, promising data and constantly evolving technology suggest that this strategy may enable precise and personalized treatment of cancer patients receiving ICI.

## 7 CONCLUSION

In this paper, the clinical trials of the three most widely studied biomarkers of liquid biopsy (CTCs, ctDNA, exosomes) in early tumor screening, tumor growth monitoring and tumor treatment are sorted out and summarized. It consolidates the current status and shortcomings of liquid biopsy development, providing an overview of the prospects and development of liquid biopsy. In conclusion, liquid biopsy holds great promise in cancer research and diagnosis. It offers a noninvasive and potentially more accurate alternative to conventional tissue biopsy, allowing for early detection, monitoring of treatment response, and personalized medicine. However, further research, standardization, and validation are needed to fully realize the potential of liquid biopsy in routine clinical practice.^202^

## AUTHOR CONTRIBUTIONS

Quan Cheng and Guodong Liu designed the manuscript. Hao Wang, Yi Zhang, and Hao Zhang drafted the manuscript and created figures. Hao Wang, Yi Zhang, Hao Zhang, Hui Cao, Jinning Mao, Xinxin Chen, Liangchi Wang, Nan Zhang, Peng Luo, Ji Xue, Xiaoya Qi, Xiancheng Dong, Quan Cheng, and Guodong Liu collected information and revised the manuscript. Quan Cheng and Guodong Liu funded and supervised the study. All authors have read and approved the final manuscript.

## CONFLICT OF INTEREST STATEMENT

All authors declare that they have no conflict of interest.

## ETHICS STATEMENT AND CONSENT TO PARTICIPATE

Not applicable.

## CONSENT FOR PUBLICATION

All authors have agreed on the contents of the manuscript.

## Data Availability

Not applicable.
